# The Quality and Accuracy of Mobile Apps to Prevent Driving After Drinking Alcohol

**DOI:** 10.2196/mhealth.5961

**Published:** 2016-08-08

**Authors:** Hollie Wilson, Stoyan R Stoyanov, Shailen Gandabhai, Alexander Baldwin

**Affiliations:** ^1^ Centre for Accident Research & Road Safety - Queensland (CARRS-Q) Institute of Health and Biomedical Innovation Queensland University of Technology Kelvin Grove Australia; ^2^ Centre for Children's Health Research Institute of Health and Biomedical Innovation Queensland University of Technology Brisbane Australia

**Keywords:** drink driving, alcohol, mobile apps, calculator, Mobile Application Rating Scale, blood alcohol content

## Abstract

**Background:**

Driving after the consumption of alcohol represents a significant problem globally. Individual prevention countermeasures such as personalized mobile apps aimed at preventing such behavior are widespread, but there is little research on their accuracy and evidence base. There has been no known assessment investigating the quality of such apps.

**Objective:**

This study aimed to determine the quality and accuracy of apps for drink driving prevention by conducting a review and evaluation of relevant mobile apps.

**Methods:**

A systematic app search was conducted following PRISMA guidelines. App quality was assessed using the Mobile App Rating Scale (MARS). Apps providing blood alcohol calculators (hereafter “calculators”) were reviewed against current alcohol advice for accuracy.

**Results:**

A total of 58 apps (30 iOS and 28 Android) met inclusion criteria and were included in the final analysis. Drink driving prevention apps had significantly lower engagement and overall quality scores than alcohol management apps. Most calculators provided conservative blood alcohol content (BAC) time until sober calculations. None of the apps had been evaluated to determine their efficacy in changing either drinking or driving behaviors.

**Conclusions:**

This novel study demonstrates that most drink driving prevention apps are not engaging and lack accuracy. They could be improved by increasing engagement features, such as gamification. Further research should examine the context and motivations for using apps to prevent driving after drinking in at-risk populations. Development of drink driving prevention apps should incorporate evidence-based information and guidance, lacking in current apps.

## Introduction

Drinking and driving remains a significant public health issue globally despite ongoing prevention efforts [1]. Alcohol intoxication is linked to slowed reaction time, difficulties in multitasking, reduced attention span, and dulled senses, which greatly reduce the ability to drive safely [2]. Australia is considered a world leader in legislation for drink driving prevention, with national random breath testing and a set 0.05 blood alcohol content (BAC) limit for open class license holders. However, there is also a distinct drinking culture, and drink driving behavior persists. Most licensed alcohol and drug users in the country admit to driving over the legal alcohol limit at some time, with over 40% reporting doing so at least twice in the last year [3]. One-third of crashes involve alcohol as a contributing factor [4] and as such, excessive alcohol use continues to be considered one of the main road safety concerns [5]. It is, therefore, necessary to utilize all available resources to increase drivers’ education and motivation to reduce drink driving behavior. Well-targeted mobile apps may offer an innovative, user-friendly, and accessible way of reducing drinking and driving.

Mobile phones are owned by 89% of Australian adults [6] who spend an average of 29 hours per month using apps [7]. In recent years, the number of mHealth apps published on iOS and Android platforms have more than doubled. There are currently more than 165,000 mHealth apps (free and paid) publicly available [8]. However, the effectiveness of health apps remains largely untested and unknown. Commonly occurring app inaccuracy, poor information quality, lack of evidence base, and lack of efficacy trials raise concerns about app effectiveness and even risks associated with app use [9,10]. Potential hazards range from user misinformation all the way to misdiagnosis of disease [11]. In-depth, systematic review and evaluation of apps in all health areas is needed to inform end users, clinicians, and developers of best practices and common problems in existing apps [12]. Thus, apps that provide calculations of BAC level, for example, should be scrutinized to ensure their quality and accuracy in providing correct guidance on readiness to drive after consuming alcohol.

An attempt at developing a systematic heuristic for the categorization and evaluation of app quality is provided by the Mobile App Rating Scale (MARS). According to the authors, high-quality apps are generally customizable, engaging, well-targeted, easy to use and navigate, and contain high-quality graphics and information [12]. The scale contains 4 objective quality subscales (19 items): engagement, functionality, aesthetics, and information quality and one subjective quality subscale (4 items). The MARS has been recently applied to determine the quality of apps for mindfulness [13], weight loss and smoking cessation [14], and heart failure symptom monitoring [15] and was therefore deemed an appropriate measurement tool for this study.

In the drink driving context, high-accuracy apps provide specific and correct information based on customizable user content in (BAC) calculators. A recent review of apps found that calculator apps tend to overestimate BAC level and provide an extremely wide variation in scores [16]. This research suggested that such calculations were based on insufficient user data (ie, height, age, times spent drinking, and so forth) and flawed calculation methods. It is also known that apps addressing alcohol use are rarely theory or evidence based [9].

A review of apps targeting drink driving has not yet been conducted, despite the need for expert review and evaluation of their accuracy and potential application. This study aimed to (1) conduct a systematic contextual review of drink driving apps, (2) use a validated app rating scale (MARS) to measure app quality, and (3) assess BAC calculators in apps for accuracy. The secondary aim was to highlight some of the best practices and potential issues in such apps as used for ecological momentary assessment and ongoing behavior change.

## Methods

### Systematic Contextual Review

Recent research into app quality highlights the necessity for systematic contextual app reviews, appropriate categorization, and expert evaluation [12]. A systematic search of apps with drink driving prevention content was conducted in June 2015 following PRISMA guidelines. The search utilized the Google “app search” filter. Searches were conducted, for the terms “drink tracker,” “alcohol tracker,” “alcohol driving,” “drink driving,” “drunk driving,” “intoxicated driving,” “DUI” (driving under the influence), “DWI” (driving while intoxicated), “BAC,” and “blood alcohol concentration.”

By default, Google returns large numbers of results. Therefore, careful scrutiny of each result page for each of the search terms was done before shortlisting and downloading all potentially-relevant apps. Initially all app titles and where necessary, app descriptions were screened. Apps were excluded if they were non–drink-driving related, duplicate, inaccessible, or not in English language. All remaining apps were downloaded and explored. Those which only measured alcohol in fluid ounces were excluded, as this measurement type is not applicable to the Australian context. Apps needed to be available in the Australian app store (though they may have been developed overseas and still applicable to the research study), as the enforced Australian BAC limit is 0.05 for open license holders. Apps related to other jurisdictions BAC limits or related detection (eg, providing information about achieving a 0.08 limit such as in the United States or how to pass sobriety tests) were excluded. Inclusion criteria were apps that either directly targeted drink driving prevention (ie, included information relating to the reading of or strategies aimed at lowering of a BAC) or apps that included information about alcohol use and its role in drink driving. Eligible apps included information about drink driving, regardless of whether drink driving prevention was the primary or secondary purpose of the app.

### App Rating

The MARS contains 23 items rated on a 5-point scale (1=inadequate, 2=poor, 3=acceptable, 4=good, and 5=excellent) or not applicable. Apple iOS apps were rated and reviewed on an iPhone 6 Plus (iOS 8.4.1) and Android apps were rated on a Samsung Galaxy Edge (Android 5.0.2). All apps were rated by 2 raters to increase reliability of results. Scores were averaged for each MARS item. Both raters underwent MARS training, as suggested by Stoyanov and colleagues [12] and followed the steps presented in the YouTube training tutorial [17]. To address information-specific items, a researcher specializing in drink driving information and behavior provided a 1-hour structured information session to each rater before app evaluation. For item 19 relating to evidence base, raters conducted a literature search in Google Scholar utilizing the app name as a search term. A thorough Internet search was also conducted for each app, including the developer website, to examine any available unpublished studies.

### Analyses

Measures of interrater reliability were conducted using the intraclass correlation coefficient (ICC) [18] on all MARS subscales and total score. A 2-way mixed effects, average measures model with absolute agreement was utilized [19]. Independent t tests were conducted to determine the differences between alcohol management apps and drink driving prevention apps, and effect sizes were calculated [20].

For apps containing BAC calculators, an assessment was conducted to determine the accuracy and similarity of their output. For this purpose, identical information was entered into all calculator apps, accounting for male or female users. Average Australian demographics used to test BAC calculators included: male, 25 years, 86 kg, 176 cm, 2 standard drinks over an hour; and female, 25 years, 71 kg, 162 cm, 1 standard drink over an hour (to improve accuracy across app calculations, where possible, 1 standard drink was equivalent to a mid-strength beer consisting of 375 mL, 3.5% alcohol). This calculation was used due to the widespread advice on the amount of standard drinks that can be consumed to stay under the 0.05 BAC limit, which is different for men (2/hr) and for women (1/hr), though the most recently released guidelines suggest that “for most adults, drinking no more than 2 standard drinks on an occasion will keep the BAC below 0.05” (p. 85) [21].

Key features were noted to provide a general overview of what could be expected across apps. The popularity and effectiveness of these features could highlight possible considerations for inclusion in the design of an app moving forward.

## Results

### Systematic Contextual Review

**Figure 1:**
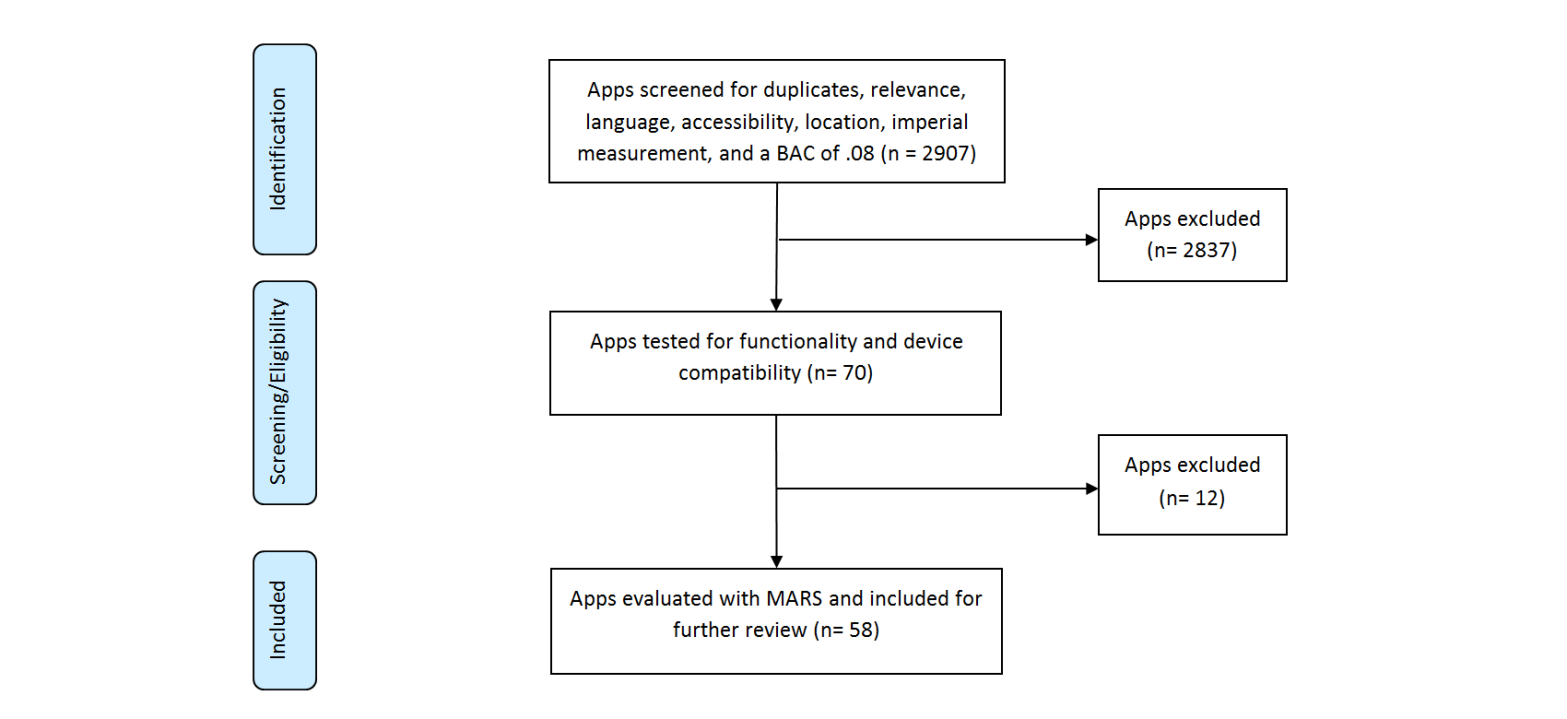
Systematic search of drink driving prevention apps selected for MARS analysis.

A total of 2907 apps were identified through keyword searches. Seventy apps were downloaded and explored. Of them, 58 were eligible for MARS evaluation at the final stage. Of these, 22 (38%) were developed by single developers and the remaining 36 (62%) were developed by institutions or businesses. The median time since last update was 16 months. The mean number of downloads for alcohol management apps was 7440 and the mean number of downloads for drink driving prevention apps was 27,266 (sourced from xyo). Figure 1 depicts the results of the systematic search.

Of these, 28 were Android and 30 were iOS apps. There were 2 core app types: alcohol management apps, containing secondary information about drink driving (n=14) and drink driving prevention apps containing Widmark-based calculators (n=44) (see Multimedia Appendix 1). These 2 app groups were separated for analysis, as they are functionally different and are aimed at different groups (alcohol management vs drink driving prevention).

### MARS Reliability

The first analysis involved examination of the internal consistency and interrater reliability of the MARS and its subscales. Independent ratings demonstrated good internal consistency (Cronbach alpha = .84) and excellent interrater reliability for the total MARS (2-way mixed ICC = 0.84, 95% CI 0.80-0.87) and for all subscales [17] (Table 1).

**Table 1:** Interrater reliability of the MARS subscales (95% CI).

MARS subscale	Intraclass Correlation Coefficient
Engagement	.78 (.64-.86)
Functionality	.84 (.71-.91)
Aesthetics	.86 (.78-.91)
Information	.80 (.40-.90)

### App Quality of Alcohol Management and Drink Driving Prevention Apps

Quality measures as detailed in MARS subscales and total mean scores were calculated to examine individual app quality and to present a comparison of the quality of the 2 types of apps (Table 2). For details of the mean app rating scores and subscale scores for all apps included in the analysis, see Multimedia Appendix 1.

**Table 2:** Comparison of MARS subscale means and standard deviations in parenthesis, between alcohol management and drink driving prevention apps.

MARS subscale^a^	Alcohol management^b^	Drink driving prevention^b^
Engagement	3.14 (0.78)	2.51 (0.70)
Functionality	3.83 (0.73)	3.57 (0.82)
Aesthetics	3.23 (0.91)	2.80 (1.03)
Information^c^	3.16 (0.74)	2.78 (0.43)
MARS mean	3.34 (0.69)	2.91 (0.57)

^a^MARS values range from 1 – inadequate to 5 – excellent.

^b^The rated versions (Multimedia appendix 1) of the apps may not be available in the App Store at the time of publication, as they may be replaced by newer versions.

^c^The information quality score excluded Item 19 of the MARS.

Independent t tests were used to compare the mean scores between alcohol management apps and drink driving prevention apps on the subscales of the MARS (engagement, functionality, aesthetics, information, and overall quality mean). There was a significant difference in the scores for alcohol management apps and drink driving prevention apps on the overall quality mean; *t* (56) = 2.31, *P*=.02, 95% CI (0.06-0.79), and d=.68 and the engagement subscale; *t* (56) = 2.88, *P*=.01, 95% CI (0.19-1.07), and d=.85. There was no significant difference in the scores for alcohol management apps and drink driving prevention apps on the functionality subscale; *t* (56) = 1.09, *P*=.28, 95% CI (−0.22 to 0.76), d=.33, the aesthetics subscale; *t* (56) = 1.37, *P*=.18, 95% CI (−0.20 to 1.04), d=.44, or the information subscale; *t* (15.87) = 1.82, *P*=.09, 95% CI (−0.06, 0.82), d=.63. For the information subscale analysis, Levene’s test indicated unequal variances (*F*=5.15, *P*=.03), so degrees of freedom were adjusted from 56.00 to 15.87. We could find no evidence that any app had been evaluated in either scientific literature or the Internet search, which is why item 19 “evidence base” was consistently rated as N/A.

### Investigation of Information Scale Items

As there are widespread misconceptions in information sources about the safety of driving after consuming alcohol [22], provision of wrong or misleading information to app users could potentially lead to poorly-informed decisions on readiness to drive. Therefore, the information section of the MARS scale was not solely presented as a mean score, but also as individual items, so that scores of the quality and quantity of information could be reviewed separately (Table 3).

**Table 3:** Comparison of the MARS information subscale items overall mean scores and standrard deviations in parenthesis between alcohol management apps drink driving prevention apps .

Information subscale item	Alcohol management (n=14)	Drink driving prevention (n=44)
Accuracy of app description	3.89 (0.84)	3.57 (0.70)
App goals	3.75 (0.91)	3.24 (0.73)
Information quality	2.61 (0.66)	2.59 (0.39)
Information quantity	3.14 (1.18)	2.27 (0.84)
Visual information	3.12 (0.98)^a^	3.16 (0.69)^b^
Credibility of the source	2.32 (0.61)	1.93 (0.41)
Evidence base	N/A	N/A
Information mean	3.16 (0.74)	2.78 (0.43)

^a^n=13 (apps rated as N/A were not included in the calculations).

^b^n=32 (apps rated as N/A were not included in the calculations).

### Calculator Accuracy Assessment

An analysis of the BAC calculators that were included in the apps was conducted to assess their accuracy. A random selection of apps with BAC calculators were included (n=35). Apps with the ability to only calculate the number of standard drinks (not time taken) or that resulted in extremely conservative BAC scores (outliers) were excluded from the analysis (n=2). The average male achieved a mean BAC of 0.03 (standard deviation = 0.01) ranging from 0.01 to 0.05, with an average time until sober of 1:48 hours. The average female achieved a mean BAC of 0.01 (standard deviation = 0.01) ranging from 0.00 to 0.05, with an average time until sober of 1:02 hours.

### App Features and Best Practice

**Figure 2:**
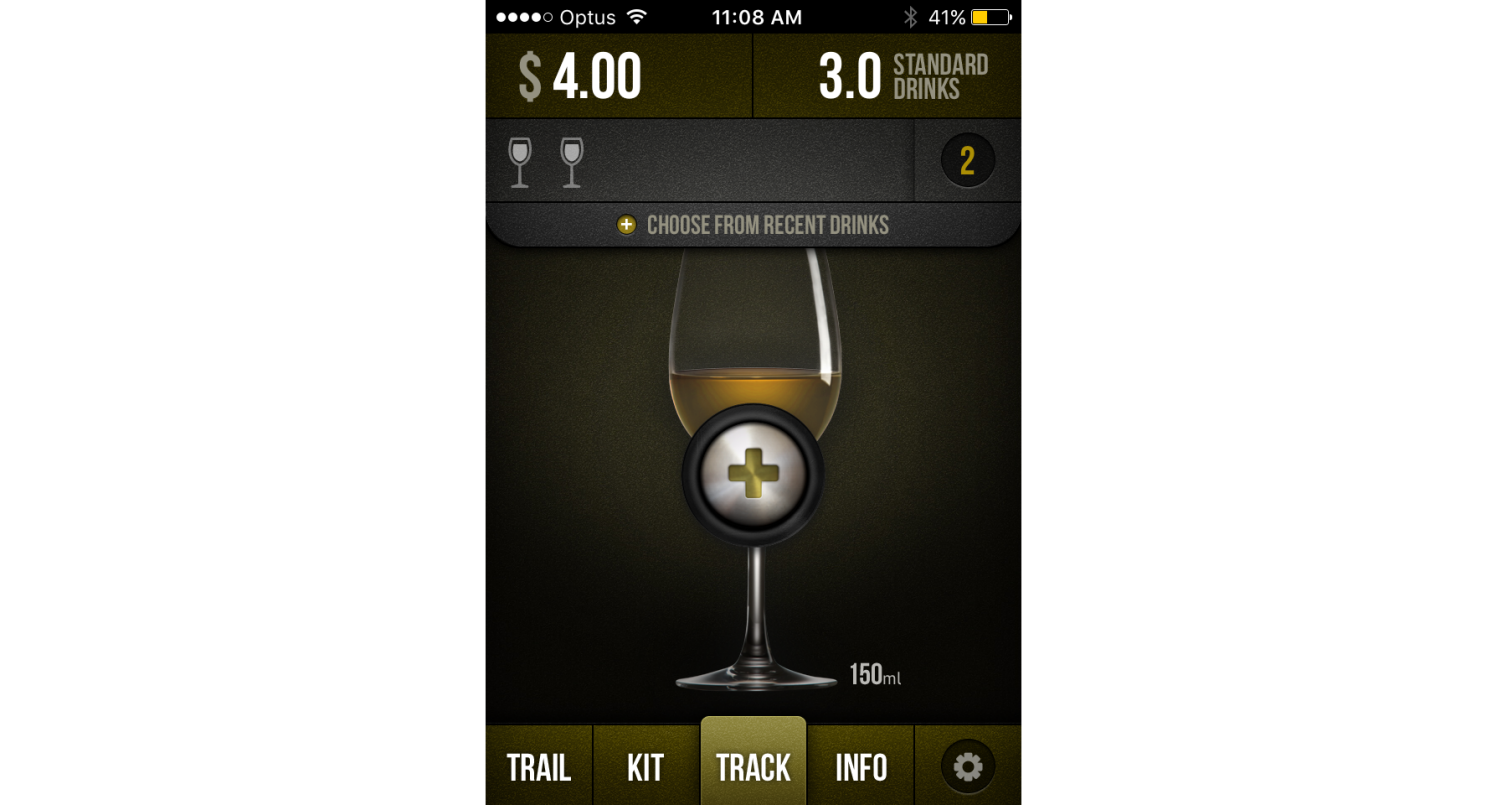
OnTrack Screenshot 1.

**Figure 3:**
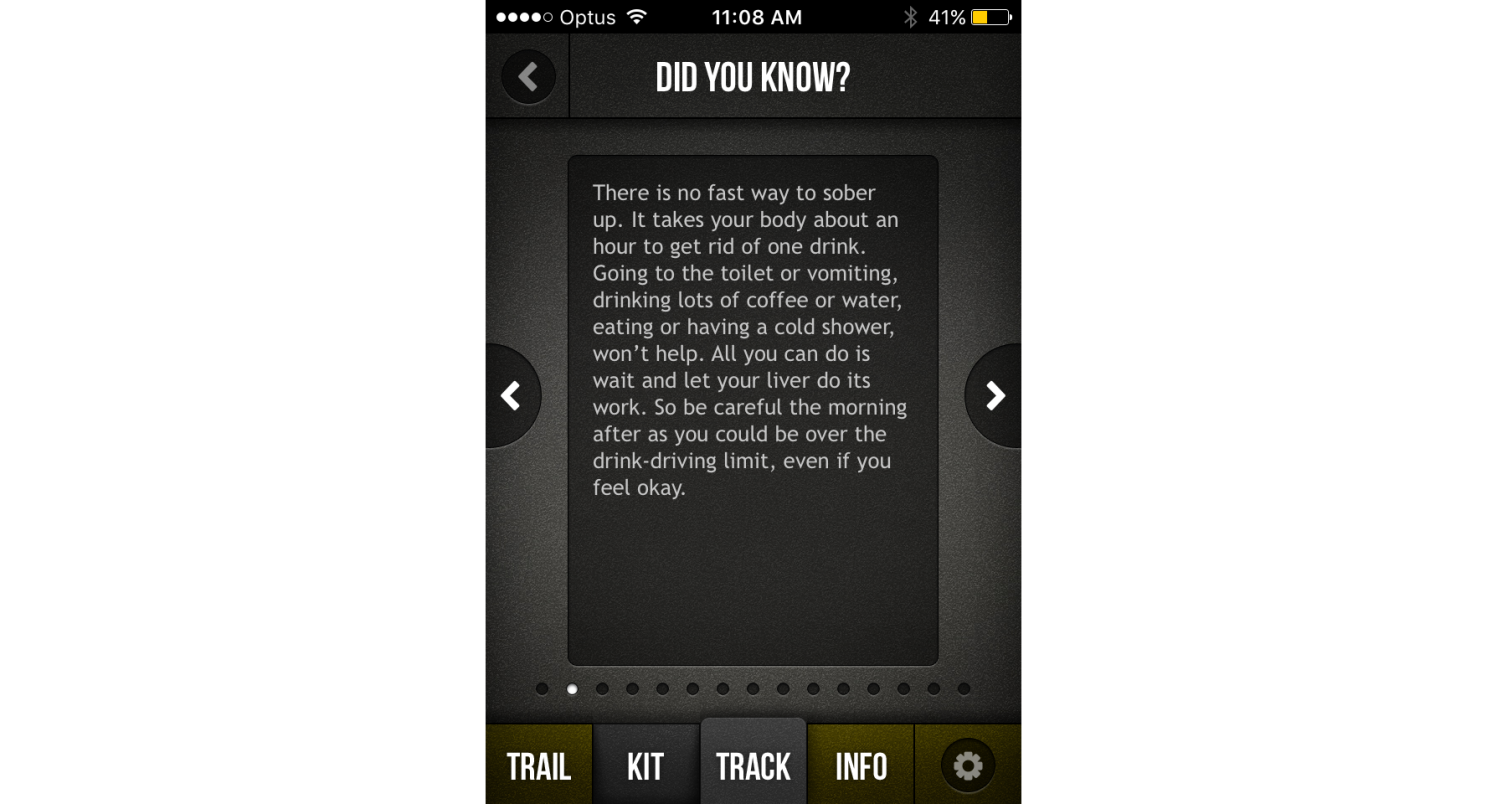
OnTrack Screenshot 2.

**Figure 4:**
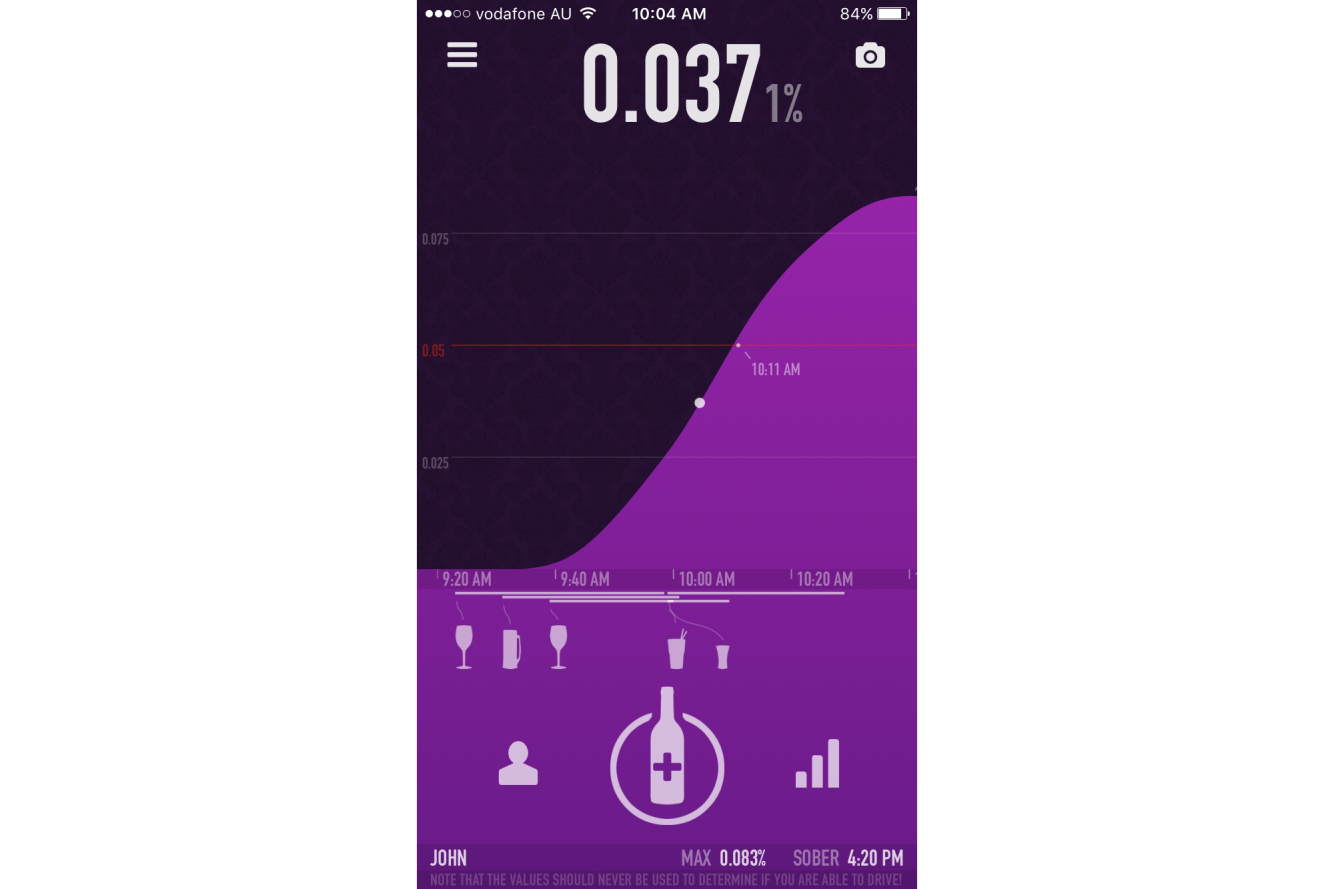
IntelliDrink Screenshot 1.

**Figure 5:**
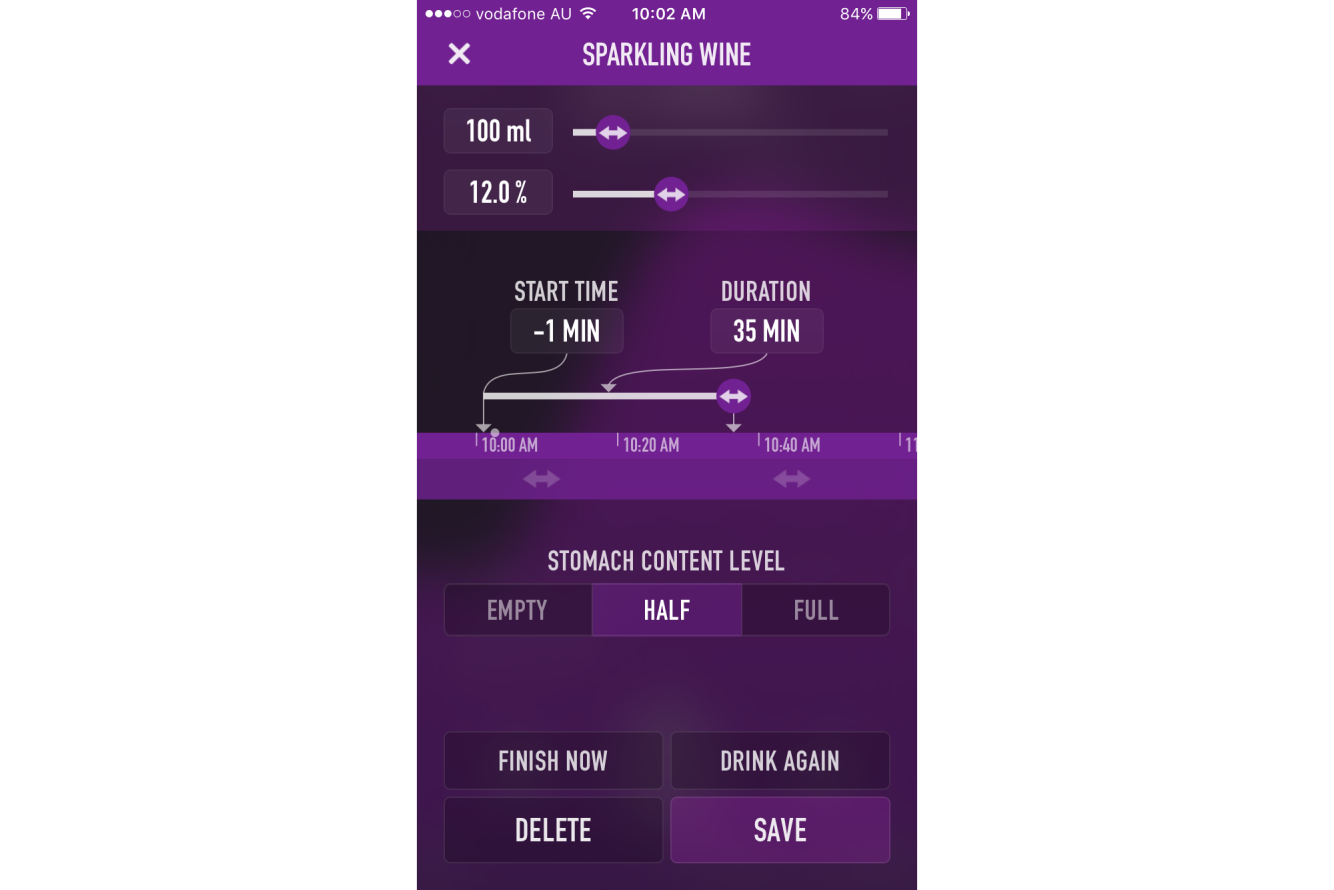
IntelliDrink Screenshot 2.

In terms of useful and engaging features, alcohol management apps generally provided links to additional research-based Web-based content, an in-app diary to be utilized for tracking, quizzes to test alcohol knowledge, and personalized feedback relating to alcohol consumption norms (local or global). As drink driving prevention apps generally aimed to provide ecological momentary assessment of readiness to drive, many of the high rating apps provided a level of personalization to improve accuracy, such as creating a user profile (gender, age, height, weight) and the ability to log multiple profiles. In terms of BAC calculation, best practice for apps involved warnings that the information provided is a guideline only, being able to input specific drink data (such as grams or percentage of alcohol), and provision of information relevant to the country of origin (eg, related to BAC level). The high-quality apps generally also contained information and links to public transport or taxi options and prompts to contact friends. Many of the lower quality apps encouraged the use of alcohol by providing features such as sharing BAC level to social media or finding local venues where alcohol is available. For screenshots of the highest rated alcohol management and drink driving prevention apps in this study, please refer to Figure 2-5.

## Discussion

### Principal Findings

Driving after the consumption of alcohol presents a significant risk, and novel strategies such as mobile apps are emerging as a potential intervention strategy for prevention and behavior change. This research is the first to explore the quality of mobile apps specifically including information and strategies in the prevention of drink driving in Australia. By conducting a systematic and contextual review of relevant apps, we were able to determine that apps containing drink driving information and intervention strategies fell into 2 categories: drink driving prevention (largely utilizing calculators for a time-until-sober calculation) and alcohol management (largely utilizing harm reduction strategies to reduce alcohol use and subsequent risk taking). The overall quality between the two app types differed as a function of significantly different engagement scores. Although drink driving prevention apps had 3.5-fold more downloads on average than alcohol management apps, they were significantly less engaging. The 2 app types identified in this research are likely to be used for different purposes. Calculator apps may be utilized for ecological momentary assessment purposes while drinking alcohol to assess level of intoxication and potentially readiness to drive, whereas alcohol management apps are likely to be used in the reduction of risky and harmful drinking. Thus, the latter is used for a broader purpose.

The quality of apps was assessed and then calculators in drink driving prevention apps were analyzed for accuracy. The key issue we found in calculator apps was their potential inaccuracy based on the formula they used. The “Widmark” formula is a predictive mathematical equation that was developed in 1932 that has largely been used forensic toxicologists to determine approximate BAC after a fatality for court proceedings [23]. There is a large and growing body of evidence that these calculations provide misleading and inaccurate information by underestimating actual BAC levels [24-26]. Replicating the findings of earlier research [16], the present study found that although calculators were largely conservative (overestimating BAC compared with national guidelines), they were inconsistent and some could lead to the provision of advice on readiness to drive when someone is still at risk of being over the legal alcohol limit, particularly for women. This is a concern particularly as the proportion of female drink drivers continues to rise [27]. Our results also confirm previous findings in the lack of evidence base and suitable evaluation for alcohol apps [9], and this should be the focus of future research to determine efficacy. In addition, while the “Widmark” formula was the most commonly noted basis for BAC calculations in the apps reviewed, a number of apps failed to indicate what formula was in use or how they arrived at their BAC value at all. This was concerning not only due to the ambiguity of app’s calculations but also in conjunction with high app downloads and positive user reviews. This suggests a user’s choice of app may be influenced by factors beyond calculation method transparency or accuracy.

On determining whether one should drive after drinking any alcohol, it should also be noted that impairment can occur at very low levels [28], supporting the argument that calculators are ambiguous and should not be used in this context. There is evidence that skills performance starts to deteriorate at levels well below a 0.05 BAC, especially in terms of divided attention and basic driving skills [29,30]. Compared with drivers with no alcohol in their system, the risk of a drink driving crash rises for drivers with a BAC of 0.05 or greater [28]. However, balancing the evidence, it would seem that having a conservative tool in which to measure potential risk could aid in decision making to avoid drink driving. The nonambiguous message remains to separate drinking from driving completely, but while BAC limits are enforced, it is unlikely that drinkers will adopt a stance that does not enable them to calculate drinks to stay under the legal alcohol limit.

Although this study provides novel results, there are also limitations that should be considered. First, only apps applicable to the Australian context were described due to consistent legislation and detection practices and further research should be conducted to determine the quality of drink driving apps in other areas. For example, sobriety apps providing guidance on subjective assessments of impairment should be the focus of research in jurisdictions where utilizing these methods of detection. Thus, the generalizability of these results to other jurisdictions is unknown.

As calculator apps are often highly simplified, there were apps that were tested where the exact volume and percentage of alcohol could not be determined (ie, the app included only a graphic of a beer, wine or spirit with no other information). For other apps, only an amount closest to 1 standard drink could be entered, thus accuracy of calculators could be skewed due to the inability to input standard drink data. However, apps included the ability to input the time taken to consume the beverage (eg, 1 standard drink consumed over 15 minutes or over 30 minutes), which should have added to the accuracy of BAC measurement. A number of apps also included the ability to indicate the degree of food consumption (eg, empty, half full, full); however, additional accuracy of such apps was not examined in detail.

Finally, due to the ever-evolving app market, with regular additions and removal of apps and updating of search algorithms in app searches, this research provides a snapshot of apps only during the study period, and app studies should be regularly updated. It must also be noted that operating system may affect the availability of older or outdated apps. Older iOS apps were often unable to function on newer versions of the operating system and thus were automatically obsolete if not updated. However, older Android apps maintained compatibility with newer OS versions and, without manual removal by the developer, have the potential for containing outdated information and content which increases the risk of negative consequences resulting from their use.

### Future Research and Development

This research has demonstrated that there are numerous apps containing information for prevention of drinking and driving. Further research needs to be conducted to determine the contexts in which these apps are used, and the motivations for engaging with them.

Engagement is a key difference in alcohol management versus drink driving prevention apps, and thus components of more engaging apps could be transferable to less engaging ones, such as interactivity (eg, providing feedback on alcohol consumption and its progressive effect throughout the session, prompting the user to slow down or increase hydration, and utilizing notifications to keep the user informed about their current state of alcohol consumption), customization (ie, the ability to change the design to keep favorite/frequent drinks at the forefront, tailored information to provide the user with their physiological traits they feel would be most beneficial such as current BAC, time until sober, number of standard drinks consumed, tally of the cost of drinks over the period of a session, etc), entertainment (eg, awarding points for good behavior and the ability to cash in the points on unlocking aesthetics features), and interest (eg, the use of animations or eye catching design elements) [31,32].

In designing an app in this context, key elements to be considered should include: motivation for use (ie, engagement strategies), context of use (ie, as a tool to predict when is the earliest time to begin driving again), reason for continued use (ie, as a tool to track drinking/alcohol consumption) unobtrusiveness (ie, should incorporate a clear clean design showing only essential information as customized to personal preference and attempt to minimize required time spent in-app), and cost (ie, the value a user will place on the functionality to justify either paying for the app or choosing a free alternative). Technologically advanced novel features could include: location based (ie, the apps recognizes user location and customizes the number of drinks offered, cost of drinks, how best to get back home, automatically launching a session, etc), smart watch integration (ie, the ability to view alcohol consumption/BAC at a moment’s notice with minimal interruption to social situations), barcode scanning of drinks (ie, more accurate information could be supplied and updated in a central database and could be much more convenient than entering specific drink information), social elements (eg, ability to track with friends, notification when a friend may require assistance or is unsafe to drive), and pre-emptive prompts (ie, information on how much alcohol may be consumed before driving may be unsafe). There is also scope to pair these apps with relevant hardware that could more accurately measure BAC such as fuel cell based breathalyzers.

### Conclusions

Most apps for drink driving prevention are not engaging, and none have as yet been tested in trials to determine their effectiveness in reducing drink driving behavior. While drink driving prevention apps are a promising countermeasure addressing risky road user behavior, they require an evidence base to ensure their quality and accuracy, and this currently does not exist.

## Appendix

Multimedia Appendix 1 Mobile apps and MARS subscale and total quality scores for both app category types.

## Abbreviations

BAC: blood alcohol content
ICC: intraclass correlation coefficient
MARS: Mobile App Rating Scale
PRISMA: Preferred Reporting Items for Systematic Reviews and Meta-Analyses

## References

[1] Martineau F, Tyner E, Lorenc T, Petticrew M, Lock K (2013). Population-level interventions to reduce alcohol-related harm: an overview of systematic reviews. Prev Med.

[2] Calhoun V, Pekar J, Pearlson G (2004). Alcohol intoxication effects on simulated driving: exploring alcohol-dose effects on brain activation using functional MRI. Neuropsychopharmacology.

[3] Papafotiou Owens K, Boorman M (2011). Evaluating the deterrent effect of random breath testing (RBT) and random drug testing (RDT) The driver's perspective.

[4] Australian Transport Council (2011). Roadsafety.

[5] Terer K, Brown R (2014). Australian Institute of Criminology.

[6] (2014). Australian Mobile Phone Lifestyle Index.

[7] (2015). Nielsen Mobile Ratings.

[8] (2016). IMS Health.

[9] Crane D, Garnett C, Brown J, West R, Michie S (2015). Behavior change techniques in popular alcohol reduction apps: content analysis. J Med Internet Res.

[10] Pagoto S, Bennett GG (2013). How behavioral science can advance digital health. Transl Behav Med.

[11] Abroms LC, Lee WJ, Bontemps-Jones J, Ramani R, Mellerson J (2013). A content analysis of popular smartphone apps for smoking cessation. Am J Prev Med.

[12] Stoyanov S, Hides L, Kavanagh D, Zelenko O, Tjondronegoro D, Mani M (2015). Mobile app rating scale: a new tool for assessing the quality of health mobile apps. JMIR Mhealth Uhealth.

[13] Stoyanov SR, Hides L, Kavanagh DJ, Zelenko O, Tjondronegoro D, Mani M (2015). Mobile app rating scale: a new tool for assessing the quality of health mobile apps. JMIR Mhealth Uhealth.

[14] Patel R, Sulzberger L, Li G, Mair J, Morley H, Shing M, O'Leary C, Prakash A, Robilliard N, Rutherford M, Sharpe C, Shie C, Sritharan L, Turnbull J, Whyte I, Yu H, Cleghorn C, Leung W, Wilson N (2015). Smartphone apps for weight loss and smoking cessation: Quality ranking of 120 apps. N Z Med J.

[15] Masterson CR, Maurer MS, Reading M, Hiraldo G, Hickey KT, Iribarren S (2016). Review and Analysis of Existing Mobile Phone Apps to Support Heart Failure Symptom Monitoring and Self-Care Management Using the Mobile Application Rating Scale (MARS). JMIR Mhealth Uhealth.

[16] Weaver ER, Horyniak DR, Jenkinson R, Dietze P, Lim MS (2013). “Let's get Wasted!” and Other Apps: Characteristics, Acceptability, and Use of Alcohol-Related Smartphone Applications. JMIR Mhealth Uhealth.

[17] Stoyanov SR Youtube.

[18] Shrout PE, Fleiss JL (1979). Intraclass correlations: uses in assessing rater reliability. Psychol Bull.

[19] Hallgren Ka (2012). Computing Inter-Rater Reliability for Observational Data: An Overview and Tutorial. Tutorials in quantitative methods for psychology.

[20] Cohen J (1988). Statistical power analysis for the behavioral sciences. 2nd edition.

[21] (2009). NHMRC.

[22] Alonso F, Pastor JC, Montoro L, Esteban C (2015). Driving under the influence of alcohol: frequency, reasons, perceived risk and punishment. Substance abuse treatment, prevention, and policy.

[23] Gullberg RG, Jones AW (1994). Guidelines for estimating the amount of alcohol consumed from a single measurement of blood alcohol concentration: re-evaluation of Widmark's equation. Forensic Sci Int.

[24] Brouwer I (2004). The Widmark formula for alcohol quantification. SADJ.

[25] Gullberg RG (2007). Estimating the uncertainty associated with Widmark's equation as commonly applied in forensic toxicology. Forensic Sci Int.

[26] Friel P, Logan B, Baer J (1995). An evaluation of the reliability of Widmark calculations based on breath alcohol measurements. J Forensic Sci.

[27] Armstrong KA, Watling H, Watson A, Davey J (2014). Profile of women detected drink driving via Roadside Breath Testing (RBT) in Queensland, Australia, between 2000 and 2011. Accid Anal Prev.

[28] Compton R, Blomberg R, Moskowitz H, Burns M, Peck R, Fiorentino D (2002). Crash rate of alcohol impaired driving.

[29] Taylor B, Rehm J (2012). The relationship between alcohol consumption and fatal motor vehicle injury: high risk at low alcohol levels. Alcohol Clin Exp Res.

[30] Moskowitz H, Fiorentino D (2000). NHTSA.

[31] Garnett C, Crane D, West R, Brown J, Michie S (2015). Identification of Behavior Change Techniques and Engagement Strategies to Design a Smartphone App to Reduce Alcohol Consumption Using a Formal Consensus Method. JMIR Mhealth Uhealth.

[32] Giroux D, Bacon S, King DK, Dulin P, Gonzalez V (2014). Examining perceptions of a smartphone-based intervention system for alcohol use disorders. Telemed J E Health.

