# Supplementary material for: The Quality and Accuracy of Mobile Apps to Prevent Driving After Drinking Alcohol
Source: JMIR Mhealth Uhealth. 2016 Aug 8;4(3):e98. doi: 10.2196/mhealth.5961 (PMC4993865; doi:10.2196/mhealth.5961)
Supplement: Multimedia Appendix 1 [file mhealth_v4i3e98_app1.pdf]

Alcohol Management Apps

	App Name	Engagement	Functionality	Aesthetics	Information	Subjective Score	Quality Mean
1	On Track with The Right Mix	4.50	4.63	4.33	4.25	4.63	4.41
2	Drinks Meter	4.20	4.25	4.17	4.33	3.50	4.24
3	DrinkControl - track drinks and alcohol expenses	3.40	4.50	4.00	3.50	2.50	3.85
4	Breathalyser	3.80	4.38	3.50	3.58	4.25	3.81
5	GottoJab - Alcohol Test	3.20	4.50	3.83	2.92	3.38	3.61
6	Drink.app - BAC Calculator & Blood Alcohol Content Gage	3.60	3.88	3.67	3.25	3.88	3.60
7	Leaf – Your Discreet Drink Tracker	3.00	3.88	4.00	3.38	2.63	3.56
8	AlcoDroid Alcohol Tracker	3.60	3.38	2.67	3.67	3.25	3.33
9	Tipple - Alcohol Tracker	2.90	4.00	3.17	3.17	2.13	3.31
10	Melange - Alcohol Tracker	2.70	4.50	3.83	2.17	2.13	3.30
11	Easy Alcohol Calculator	2.90	3.50	2.00	2.92	3.13	2.83
12	Promille - Blood Alcohol Tracker	2.10	2.38	2.33	3.10	1.88	2.48
13	Alcohol Optimizer	2.30	2.50	2.00	2.25	2.13	2.26
14	Simple Blood Alcohol Content	1.80	3.38	1.67	1.75	2.50	2.15

Drink Driving Prevention Apps

	App Name	Engagement	Functionality	Aesthetics	Information	Subjective Score	Quality Mean
1	IntelliDrink - Blood Alcohol Content (BAC) Calculator	4.20	4.38	5.00	3.50	4.38	4.27
2	Wise Drinking: Let's be Smart by Pernod Ricard	4.50	4.38	4.00	3.67	4.88	4.14
3	alcCalc: Estimates and Displays the Alcohol Decomposition, the Time You'll Sober Up and the BAC in Realtime.	3.30	4.13	5.00	3.00	4.00	3.86
4	BAC Alcohol Calculator	3.70	3.88	4.50	3.25	3.88	3.83
5	instaBAC	3.10	3.75	4.33	3.08	3.25	3.57
6	Drinker - A BAC (Blood Alcohol Content) calculator	3.10	4.38	3.67	2.92	3.00	3.51
7	Alcoholmeter	2.00	5.00	4.17	2.42	2.00	3.40
8	WhatsAlc - BAC Calculator	2.40	4.00	4.33	2.83	2.75	3.39
9	Live Blood Alcohol Content Calculator - Lite	2.70	4.63	3.33	2.83	2.13	3.37
10	Drink It Smart	2.40	4.75	3.83	2.50	1.88	3.37
11	Alcohol Unit Wheel	1.80	4.63	3.83	2.83	1.88	3.27
12	Gage – Breathalyzer | BAC Calculator | Blood Alcohol Content Level | Drink Counter | Drunk	2.60	3.38	4.33	2.67	2.50	3.24

	App Name	Engagement	Functionality	Aesthetics	Information	Subjective Score	Quality Mean
	Test | Drinking Timer | Sobriety Tracker						
13	DrinkTracker Breathalyzer	2.60	3.75	3.17	3.40	3.88	3.23
14	Breathalyzer (Ethylometer)	2.00	4.75	4.00	2.10	3.13	3.21
15	Alcohol Calculator	3.10	3.88	3.00	2.60	3.13	3.14
16	DrinkLess Free	3.20	3.63	2.67	3.08	3.63	3.14
17	Shot Clock - Drink Calculator	2.60	4.00	3.17	2.80	3.25	3.14
18	Ask 4 Alcocheck	2.00	4.00	3.17	3.33	3.25	3.13
19	C2ALC - Blood Alcohol Content Calculator	2.40	4.13	3.17	2.75	1.88	3.11
20	Fit To Drive?	3.30	3.50	2.50	2.92	2.13	3.05
21	Drink Companion	3.30	3.13	2.00	3.42	3.13	2.96
22	Alcohol Monitor	2.80	3.75	2.00	3.00	2.00	2.89
23	Blood Alcohol BAC	2.90	3.50	2.00	3.08	3.00	2.87
24	Blood Alcohol Calculator	3.20	2.38	2.17	3.67	2.38	2.85
25	Am I drunk?	2.70	3.63	2.17	2.88	2.63	2.84
26	Alcometr	2.10	3.63	2.67	2.75	1.75	2.79
27	Breathalyzer	2.10	4.00	2.33	2.70	3.13	2.78
28	Blood Alcohol Concentration Calculator by AIMapps (2015)	2.70	3.38	2.33	2.67	2.00	2.77
29	iBreathalyser	1.70	4.38	2.17	2.58	1.25	2.71
30	iDrinkSmarter	2.50	3.13	2.17	2.75	2.13	2.64
31	Alcohol Monitor Pro – BAC Calculator	2.80	3.00	1.83	2.75	1.75	2.60

	App Name	Engagement	Functionality	Aesthetics	Information	Subjective Score	Quality Mean
32	DrunkDroid	2.00	3.50	1.83	2.45	1.88	2.45
33	myBAC	1.70	3.88	1.50	2.70	2.13	2.44
34	DrinkBuddy	2.30	3.00	1.50	2.92	1.63	2.43
35	BLOOD ALCOHOL CALCULATOR PRO	1.60	3.38	1.83	2.70	1.75	2.38
36	Alcohol Level Calculator	1.80	3.13	1.83	2.63	1.50	2.35
37	Can I Drive Yet?	1.70	2.50	1.83	3.00	2.00	2.26
38	Drunk Too Much?	2.30	2.38	2.17	2.08	2.00	2.23
39	Blood Alcohol Content Calc 2.0	2.00	3.38	1.67	1.88	1.88	2.23
40	Over The Limit Blood Alcohol Estimator	2.00	2.88	1.83	2.20	1.38	2.23
41	Alcohol Calculator	2.20	2.50	1.50	2.50	1.75	2.18
42	Alcotester Calculator	1.40	2.50	2.83	1.75	3.13	2.12
43	Drunk-O-Meter	2.20	1.00	2.00	2.58	1.50	1.95
44	Alcohol. Breathalyzer test	1.40	2.00	2.00	2.33	1.88	1.93
